# Gyejigachulbutang (Gui-Zhi-Jia-Shu-Fu-Tang, Keishikajutsubuto, TJ-18) in Degenerative Knee Osteoarthritis Patients: Lessons and Responders from a Multicenter Randomized Placebo-Controlled Double-Blind Clinical Trial

**DOI:** 10.1155/2020/2376581

**Published:** 2020-10-28

**Authors:** Myung Kwan Kim, Jungtae Leem, Young Il Kim, Eunseok Kim, Yang Chun Park, Jae-Uk Sul, Hee-Geun Jo, Sang-hoon Yoon, Jeeyong Kim, Ju-Hyun Jeon, In Chul Jung

**Affiliations:** ^1^Department of Acupuncture and Moxibustion Medicine, College of Korean Medicine, Daejeon University, 62, Daehak-ro, Dong-gu, Daejeon 34520, Republic of Korea; ^2^Chung-Yeon Central Institute, 64, Sangmujungang-ro, Seo-gu, Gwangju 61949, Republic of Korea; ^3^Research and Development Institute, CY Pharmaceutical Co., Ltd., 340, Nonhyeon-ro, Gangnam-gu, Seoul 06227, Republic of Korea; ^4^Division of Respirtory System, Department of Internal Medicine, College of Korean Medicine, Daejeon University, 62, Daehak-ro, Dong-gu, Daejeon 34520, Republic of Korea; ^5^Chung-Yeon Korean Medicine Hospital, 64, Sangmujungang-ro, Seo-gu, Gwangju 61949, Republic of Korea; ^6^Department of Neuropsychiatry, Daejeon Korean Medicine Hospital of Daejeon University, 75, Daedeok-daero 176 beon-gil, Seo-gu, Daejeon 35235, Republic of Korea

## Abstract

**Background:**

Gyejigachulbutang (GUI-ZHI-JIA-SHU-FU-TANG, GCB) is an herbal formula widely prescribed in traditional East Asian medicine practice for arthritis and muscle pain. We evaluated the efficacy and safety of GCB for degenerative knee osteoarthritis (KOA).

**Methods:**

Eighty patients with KOA were randomly assigned to the GCB group or the placebo group in a 1 : 1 ratio in two Korean medicine hospitals. Patients took GCB or placebo three times a day for 4 weeks. Primary outcome was the change in the visual analogue scale (VAS) score for knee pain from baseline to 4^th^ week. Secondary outcomes were the change in the VAS score from baseline to 2^nd^ week and 8^th^ week, Korean Western Ontario and McMaster Universities Osteoarthritis Index (K-WOMAC), European Quality of Life Five Dimensions questionnaire (EQ-5D), and safety.

**Results:**

There was no significant difference between the compared indicators of the GCB and placebo groups. However, in subgroup analysis, GCB was effective for subjects with a BMI lower than 25 kg/m^2^. The dose of pain medication was significantly lower in the GCB group than in the placebo group after four weeks (*p*=0.016). There were no serious adverse events in the GCB group.

**Conclusions:**

GCB was not effective in primary outcome analysis. In exploratory subgroup analysis, GCB might be effective for individuals with BMI lower than 25 kg/m^2^ for the treatment of degenerative KOA. GCB may also help reduce the consumption of pain medication. Furthermore, research is required for our hypothesis. This trial is registered with KCT0003024.

## 1. Introduction

Degenerative knee osteoarthritis (KOA) is the most common degenerative disease in adults [1]. The main symptoms are knee pain, dysfunction, swelling, and crepitation during exercise [2]. Radiological studies may reveal loss of articular cartilage, structural deformation of knee joints, and irregularity of articular surfaces [3]. The prevalence of KOA in patients over 50 years old in South Korea is high: 21.1% for men and 43.8% for women. The prevalence is higher in women, and it increases with age [4]. Pharmaceutical treatments include nonsteroidal anti-inflammatory drugs, intraarticular hyaluronate injection, intraarticular steroid injection, and short-term narcotic analgesics [5, 6]. NSAIDs were effective regardless of age, sex, radiographic KOA severity, and disease duration [6]. However, the use of drugs is limited due to side effects such as cardiovascular problems, liver failure, renal failure, gastrointestinal bleeding, and cartilage weakening [7]. Nonpharmaceutical treatments for degenerative arthritis include exercise, manual therapy, taping, and acupuncture; however, some patients show only minimal response to the treatments [5]. Recently, growth factor injections, platelet-rich plasma injections, arthroscopic partial meniscectomy, and valgus-producing proximal tibial osteotomy have been a trend in practice, although according to the Journal of the American Academy of Orthopedic Surgeons, their effectiveness is inconclusive or limited [8]. Therefore, safe and effective new treatments are needed for managing degenerative KOA. Several integrative interventions such as bromelain [9], sesame oil [10], moxibustion [11], and yoga [12] are widely used in clinical practice. GCB is a traditional herbal formula widely prescribed in East Asia for common cold, arthritis, and muscle pain [13]. Recently, research on the effects of GCB on postherpetic neuralgia [14], chemotherapy-induced neuropathy [15], rheumatoid arthritis [16], neuropathic pain in dental clinics [17], and degenerative arthritis [18] has been conducted. GCB is known to have anti-inflammatory, analgesic, and edema-reducing effects [19]. GCB is potentially useful in treating KOA, but there has not been any well-designed double-blind clinical trial showing the effectiveness of GCB. Based on a previous study [20], we developed a patient and assessor-blind placebo-controlled clinical trial protocol to evaluate the efficacy and safety of GCB in degenerative KOA.

## 2. Materials and Methods

### 2.1. Trial Design and Study Setting

**Figure 1:**
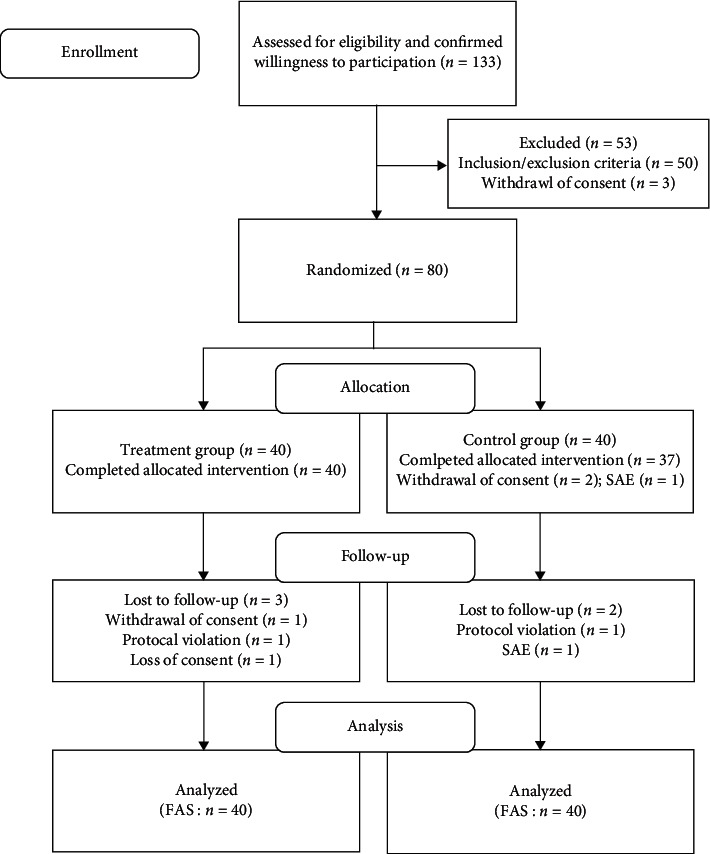
Patient flowchart.

This randomized placebo-controlled double-blind clinical trial was conducted from August 2018 to May 2019 at the Daejeon Korean Medical Hospital of Daejeon University and the Chung Yeon Korean Medical Hospital in the Republic of Korea. Eighty patients were enrolled in this study and randomly allocated to the GCB or the placebo group in a 1 : 1 ratio. The study flowchart and trial design are shown in Figure 1 and Table 1, respectively. The recommended items for a clinical trial protocol and related documents are presented according to the SPIRIT 2013 checklist [21]. We have published the clinical trial protocol previously [20].

The trial was approved by the Institutional Review Boards (IRBs) of both Korean medical hospitals (DJDSKH-18-DR-10 and CYIRB-2018-04-002). This study has been registered with the Clinical Research Information Service (CRIS), which is one of the primary registrars of the World Health Organization's (WHO) International Clinical Trial Registration Platform (KCT0003024). The study adhered to the specifications of the Helsinki Declaration (2013).

### 2.2. Recruitment

Two Korean medical hospitals located in the Republic of Korea, namely, the Daejeon Korean Medical Hospital of Daejeon University in Daejeon and the Chung Yeon Korean Medical Hospital in Gwangju, recruited 56 and 24 outpatients, respectively, in a clinical practice setting. Patients were recruited through each hospital's online homepage, bulletin boards, local newspapers, and public transportation billboards. The following analyses were conducted for patients who volunteered to participate in this trial: (a) demographic surveys, (b) vital sign measurements, (c) medical and therapeutic history, use of drugs, and treatment expectation survey [22], (d) knee X-ray, (e) blood tests, (f) electrocardiogram, and (g) additional pregnancy tests for fertile women.

#### 2.2.1. Eligibility Criteria: Inclusion Criteria

(1) Patients over 40 years old. (2) Patients who attained a VAS score higher than 30 mm for knee pain during daily life. (3) Patients who attained Grade 2 or higher on the Kellgren–Lawrence Grading Scale. (4) Patients who voluntarily decided to participate and signed the written informed consent form after receiving full explanation of the research objectives and processes.

#### 2.2.2. Eligibility Criteria: Exclusion Criteria

(1) Patients with severe knee trauma in the last 6 months. (2) Patients with a history of knee surgery or planning for surgery within the research period. (3) Patients who received steroid injection treatment within the last 3 months or hyaluronic acid injection treatment within the last 6 months. (4) Patients who received acupuncture, pharmacoacupuncture, or herbal medicine treatment for knee pain relief within the last 1 month. (5) Erythrocyte sedimentation rate (ESR) >40 mm/h or rheumatoid factor >20 U/mL on screening examination. (6) Patients with musculoskeletal problems that caused more severe pain in other parts of the body than in the knees. (7) Patients who had an uncontrolled heart condition such as angina or congestive heart failure, liver function abnormality (alanine aminotransferase or aspartate aminotransferase levels, 40 IU/L or higher), kidney function abnormality (creatinine level outside the range of 0.5–0.9 mg/dL and the blood urine nitrate level outside the range of 6–20 mg/dL), systolic blood pressure greater than 180 mmHg, or diastolic blood pressure greater than 100 mmHg. (8) Patients who were pregnant, nursing, or diagnosed with malignant tumors. (9) Patients who had genetic disorders such as galactose intolerance, Lapp lactase deficiency, or glucose-galactose malabsorption. (10) Patients who had a significant neuropsychiatric history or who were ill from neuropsychiatric disease. (11) Patients who were judged unsuitable for clinical trial participation by the principal investigator. (12) Patients who participated in another clinical trial during the last 3 months.

#### 2.2.3. Eligibility Criteria: Withdrawal Criteria

(1) Violation of inclusion and exclusion criteria. (2) Inability to continue participation due to serious adverse events. (3) Acute systemic reaction (allergy and shock) due to a clinical trial drug. (4) Manifestation of the side effects of the rescue medicine (acetaminophen), including shock (anaphylaxis symptoms), hematologic disorder (thrombocytopenia, granulocytopenia, hemolytic anemia, methemoglobinemia, platelet hypofunction, and cyanosis), hypersensitivity (facial swelling, dyspnea, sweating, hypotension, and shock), digestive system disorder (nausea, vomiting, poor appetite, gastrointestinal bleeding, digestive ulcer, and perforation), skin disorder (rash, allergic reaction, Stevens–Johnson syndrome, and Lyell's syndrome), or other disorders (chronic liver necrosis, acute pancreatitis, chronic hepatitis, and kidney toxicity). (5) Systemic diseases were not found during the screening test. (6) Surgery or hospitalization due to an accident or other illness. (7) Refusal to participate in the clinical trial. (8) Indication for conventional therapy due to worsening of knee pain. (9) Principal investigator judgment that an unavoidable reason should bar patient from participating in the study.

### 2.3. Randomization, Allocation Concealment, and Blinding

Randomization was performed by an independent biostatistician using the Strategic Applications Software (SAS) ® Version 9.4 (SAS instance. Inc., Cary, NC). Patients were randomly assigned on a 1 : 1 ratio to the GCB and placebo control groups. We did not use the block randomization method. An independent and blinded clinical research coordinator (CRC) enrolled and allocated participants according to a random number table. Random allocation tables were kept in a locked cabinet and provided to the pharmaceutical company to ensure that GCB and placebo were equally packaged, and the patients and evaluators were blinded to them. In our study, the CRC, investigator (physician), participants, assessor, and pharmacist were blinded.

### 2.4. Intervention

#### 2.4.1. Study Schedule

Table 1 shows the schedule of this study. This trial consisted of screening, treatment, and follow-up phases. On the screening visit, each participant was requested to voluntarily sign a written informed consent form before taking part in the study. Subsequently, the investigator conducted demographic surveys and medical examinations. Participants who fulfilled the eligibility criteria were scheduled for another visit within 2 weeks of visit 1. During visit 1, baseline assessments were conducted, and participants were randomized into the GCB or the placebo group. GCB and placebo was initiated for 4 weeks. During visit 2, medication was administrated, and evaluation was performed. The treatment phase was completed at visit 3. After 4 weeks from visit 3, additional follow-up evaluation was performed at visit 4.

#### 2.4.2. Clinical Trial Drug (GCB and Placebo)

GCB was manufactured by Tsumura and Co. (Tokyo, Japan). 7.5 g of GCB extract granules contain 3.75 g of additives and 3.75 g of dried extract of the following mixed crude drugs: Cinnamomi Cortex (桂皮), 4.0 g; Paeoniae Radix (芍藥), 4.0 g; Atractylodes Lancea Rhizome (蒼朮), 4.0 g; Zizyphi Fructus (大棗), 4.0 g; Glycyrrhizae Radix (甘草), 2.0 g; Zingiberis Rhizoma (生薑), 1.0 g; and Aconiti Radix Processa (附子), 0.5 g. These raw ingredients were extracted and concentrated to 2.5 g per pack (Table 2). The dosage was three times a day, taken 30 minutes after meals (7.5 g). The placebo drug was manufactured by Kyungjin Pharmaceutical and Co. (Icheon, Republic of Korea) following the Korean Good Manufacturing Practice standards. The placebo drug was composed of the caramel coloring agent, lactose, and corn starch. The placebo drug was similar in shape, color, taste, and smell to GCB. Both GCB and placebo drugs were packaged and labeled by Kyungjin Pharmaceutical and Co. using the random allocation table.

#### 2.4.3. Concomitant Treatment

In principle, all patients were prohibited from using traditional medical interventions (including acupuncture, moxibustion, herbal medicine, and cupping), conventional medications, injections, surgery, physical therapy, manual therapy, and exercise therapy to improve knee pain. Thus, in case the pain in the knee became unbearably severe during the period of participation in the clinical research, a tablet of acetaminophen (SAMNAM Pharm. Co. Ltd., 500 mg) was provided. In addition, concomitant intervention for treatment of other diseases or adverse events that would not affect the results of this trial was allowed. The research coordinator conducted a survey on concomitant treatments at every visit and confirmed that nobody underwent any prohibited concomitant treatment other than the rescue medicine.

#### 2.4.4. Rescue Medication

Acetaminophen (maximum daily dose of 3000 mg or less, six tablets per day, 500 mg/tablet) was administered as a rescue medication and taken only when the pain was unbearable. The total amount of rescue medication consumption was recorded at each visit.

### 2.5. Outcomes

#### 2.5.1. Primary Outcome

As pain is the most common complaint of degenerative arthritis, we selected the VAS as the primary outcome to assess pain severity [23]. The primary outcome of our study changed between baseline and 4 weeks on the visual analogue scale (VAS). The VAS score evaluates a person's pain intensity level. In our trial, the participants are asked to place a mark on a 100 mm horizontal line with the question “how much pain did you have during the last 3 days.” The beginning of the line illustrates “no pain,” and the end of the line indicates the “worst imaginable pain.” To extract the outcome value, the investigator measures the distance in millimeters between “no pain” and the marked point by the participant. To exclude the rescue medicine effect, the patient visited the hospital without taking any rescue medicine on the day of the evaluation.

#### 2.5.2. Secondary Outcomes: Pain

Mean change in the VAS from baseline to 2^nd^ week and from baseline to 8^th^ week.

#### 2.5.3. Secondary Outcomes: Disability

The validated Korean version of the Ontario and McMaster University Osteoarthritis Index (K-WOMAC) was used to evaluate disability associated with joint pain, stiffness, and functional status in the knees during the last 48 h [24, 25]. The differences between the K-WOMAC scores from baseline to the 2^nd^, 4^th^, and 8^th^ weeks of both groups were assessed. The K-WOMAC consisted of 24 questions (five about pain, two about stiffness, and 17 about physical functions), and that could be completed within less than 5 minutes. A total K-WOMAC score of 96 points or higher represented a poor status.

#### 2.5.4. Secondary Outcomes: Quality of Life

The European Quality of Life Five Dimensions questionnaire (EQ-5D) is a valid and reliable self-reporting questionnaire that measures a patient's health status for clinical and economic appraisal using a Likert scale and VAS [26, 27]. The changes in the EQ-5D scores from baseline to the 2^nd^, 4^th^, and 8^th^ weeks of both groups were compared.

#### 2.5.5. Secondary Outcomes: Global Assessment

The Patient Global Impression of Change (PGIC) is a valid outcome measure that is based on a seven-point Likert scale. The scale comprises “much better,” “better,” “somewhat better,” “no change,” “somewhat worse,” “worse,” and “much worse” [25]. “Much better” is rated as 7 points and “much worse” as 1 point on the PGIC. With this scale, participant responses are dichotomized into two: those that have “improved” (ratings 5 to 7) and those who have “not improved” (ratings 1–4). The change in the PGIC score and proportion of “improved” patients between the two groups were assessed at week 4 (visit 3) and week 8 (visit 4).

### 2.6. Safety and Adverse Effects

To assess the safety of the GCB, a blood test and EKG were carried out at baseline and 4 weeks. Safety was assessed by analysis of adverse events (AEs). Patients were asked to report all AEs, and cases with significant symptoms were assessed through detailed screening during the study.

### 2.7. Sample Size

Assumptions for sample size estimation were based on the results of the study by Tubach et al. [28]: change from baseline VAS = 17.9 mm, SD = 22 mm. We considered a 1 : 1 allocation, alpha = 0.05%, power = 0.9, and 20% drop out to estimate a size of 40 patients for each group. Eighty patients were required in total (56 from Daejeon Korean medicine hospital and 24 from Chung Yeon Korean Medical Hospital).

### 2.8. Statistical Methods

All statistical analyses were based on two-sided tests, a significance level of 5%, and a 95% confidence interval (CI). SAS® Version 9.4 (SAS institute. Inc., Cary, NC) was used. The primary outcome analysis was performed using analysis of covariance (ANCOVA) with the week 4 VAS score, using the baseline VAS score as covariates. We also conducted several exploratory analyses. However, as they are just exploratory analysis, we did not calculate the sample size and adopt correction for multiple comparison. In the secondary outcome analysis, we used ANCOVA for continuous data and Fisher's exact test for categorical data. For the missing data, the multiple imputation method was used.

## 3. Results

### 3.1. Patients Recruitment

Of 133 patients who visited the hospitals, 80 patients were randomly allocated at a 1 : 1 ratio to the GCB or placebo control group; 50 patients who violated the inclusion and exclusion criteria and 3 patients who refused consent were excluded. Of the 80 patients who were initially enrolled in the study, 40 were assigned to each of the GCB and placebo groups. Seventy-two patients completed the study, and eight dropped out. Of the eight who dropped out, three were in the GCB group and five were in the placebo control group. Of the three GCB group dropouts, one withdrew consent, one violated the protocol, and one went out of contact. Of the five placebo control group dropouts, two withdrew consent, two had severe adverse events (SAE), and one violated the protocol (Figure 1).

### 3.2. Baseline Characteristics of Patients

Table 3 shows the baseline characteristics of patients. There were no significant differences in age, height, weight, body mass index (BMI), the existence of job, exercise status, exercise time, Kellgren–Lawrence grade (K-L grade), morbidity period, and treatment expectancy scale point between the GCB group and the placebo group.

### 3.3. Outcome Evaluation

#### 3.3.1. Primary Outcome

The average VAS score reduced by 3.93 in the GCB group, and it was statistically significant (*p*=0.0196). On the contrary, it increased by 1.12 in the placebo control group; this was not statistically significant. There was no statistically significant difference between the two groups (Table 4). We used the value of partial eta-square for effect size. The effect size of VAS at week 4 was 0.021, which indicated a normal effect size. The effect size interpretation criteria of partial eta-square were as follows: 0.01 is small, 0.06 is normal, and 0.14 is large.

There are several exploratory secondary outcomes in our study. As they are just exploratory outcome variables, we did not calculate the sample size or adopt multiple comparison correction.

#### 3.3.2. Secondary Outcomes: Pain

From baseline to 2^nd^ and 8^th^ weeks, the average VAS score reduced by 5.4 and 10.78, respectively, in the GCB group, and the difference was significant (*p* ≤ 0.0001, 0.0174). The placebo control group had reductions of 7.54 and 10.13, respectively, and the differences were also significant (*p*=0.0061, 0.0109). There was no significant difference between the two groups (Table 4).

#### 3.3.3. Secondary Outcomes: Disability

From baseline to 2^nd^ and 4^th^ weeks, the average K-WOMAC score reduced by 2.93 and 2.83, respectively, in the GCB group, and the difference was significant (*p*=0.0064, 0.0054). However, in the 8^th^ week, the K-WOMAC score reduced by 3.61 (*p*=0.1768), and the difference was not significant. From baseline to 2^nd^ and 4^th^ weeks, the average K-WOMAC score reduced by 2.97 and 2.96, respectively, in the placebo group, and the difference was not significant. However, in the 8^th^ week, the score reduced by 8.95, and the difference was significant (*p*=0.0020). There was no significant difference between the two groups (Table 4).

#### 3.3.4. Secondary Outcomes: Quality of Life

During the study period, there was no significant difference between the assessments of the GCB group. In the placebo group, there was no significant difference between outcomes in 2^nd^ and 4^th^ weeks. However, in the 8^th^ week, the EQ-5D score increased by 0.057, and the difference was significant (*p*=0.0337). There was no significant difference between the two groups (Table 4).

#### 3.3.5. Analysis of Trends over Time

RM-ANOVA was used to analyze trends over time. VAS, K-WOMAC, and EQ-5D changed significantly with time (*p*=0.0004, 0.0021, 0.0119). However, the group × week interaction did not change significantly.

#### 3.3.6. Secondary Outcomes: Global Assessment

From baseline to 4^th^ and 8^th^ weeks, we compared those who responded “improved” (ratings 5 to 7) and “not improved” (ratings 1 to 4) in the GCB and placebo groups. The “improved” responses increased from 47.5% to 52.6% in the GCB group and 59.5% to 65.7% in the placebo group. However, there was no significant difference between the two groups.

#### 3.3.7. Exploratory Subgroup Analysis (Post Hoc Analysis): BMI

BMI is an important factor in knee pain; the higher the pain, the higher the prevalence of knee arthritis [29]. We know from experience that GCB does not work well in obese patients. Therefore, subgroup analysis was performed based on BMI 25, which is the general standard for overweight [30]. There were 43 participants with a BMI of 25 or higher and 37 participants with BMI lower than 25. In participants with a BMI of 25 or higher, the VAS score reduced significantly in both groups (*p*=0.0062, 0.0093). However, there was no significant difference between the two groups. In participants with a BMI lower than 25, the VAS score continuously reduced throughout the study period in the GCB group, and the difference was significant (*p* < 0.0001). The VAS score of the placebo group reduced significantly in 2^nd^ and 8^th^ weeks (*p* < 0.0001). In the 4^th^ week, compared to baseline, the GCB and placebo groups demonstrated a significant difference (*p*=0.0239) (Table 5).

#### 3.3.8. Safety Evaluation

During the study, a total of 41 adverse events occurred; 24 occurred in the GCB group and 17 in the placebo control group. Of the 24 adverse events occurring in the GCB group, six adverse events were likely to have been caused by clinical trial drugs (abdominal distension, diarrhea, dry mouth, increased blood pressure, increased alanine aminotransferase, and abdominal discomfort), and 18 were unrelated. Mild abdominal distension, diarrhea, dry mouth, and abdominal discomfort, among others, were observed. Of the 17 adverse events in the placebo group, three were possibly related to the clinical trial drug (abdominal discomfort, hypertension, and palpitation), and 14 were considered unrelated. Two severe adverse events of hypertension and back pain occurred in the placebo group, but they recovered. On blood testing, a significant decrease in the platelet level was reported in one participant in the GCB group in the 4^th^ week, but it was within normal limits. There were no significant differences between the safety indicators of the two groups.

#### 3.3.9. Blinding Maintenance

In the 4^th^ week of the trial, there was a significant difference between the GCB group and placebo group (*p*=0.0104). While the GCB group accounted for 2.5% of those who thought they were taking placebo alone, the placebo group accounted for 37.8% of those who thought they were taking GCB. The new blind index was used for blinding assessment [31]. In the interpretation of the result, if 0 is within 95% of the confidence interval, blinding is well maintained. The figures (average, 95% CI) for the new blind index of the GCB group and placebo group were 0.350 (0.187, 0.513) and −0.135 (−0.385, 0.115), respectively. In our study, the placebo group participants randomly guessed their allocation group (Table 6).

In week 4, total and mean rescue medicine consumption was significantly lower in the GCB group than in the placebo group after the 4^th^ week. In the GCB group, 17 participants took 92 rescue medicines in total. In the placebo group, 15 participants took 176 rescue medicines in total (Table 7).

#### 3.3.10. Medicine Compliance (Post Hoc Analysis)

In the GCB group, the average compliance was 96.56 at the 2^nd^ week and 95.18 at the 4^th^ week, while in the placebo group, it was 97.06 at the 2^nd^ week and 93.28 at the 4^th^ week. There was no significant difference between the two groups.

## 4. Discussion

GCB is a traditional herbal formula widely used in traditional East Asian medicine (TEAM), and it is composed of Cinnamomi Cortex, Paeoniae Radix, Atractylodes Lancea Rhizome, Zizyphi Fructus, Glycyrrhizae Radix, Zingiberis Rhizoma, and Aconiti Radix Processa. GCB has been widely used for several conditions such as influenza, common cold, arthritis, and muscle pain in clinical practice [13].

In this 8-week randomized double-blind placebo-controlled clinical trial, we evaluated the efficacy and safety of GCB as a treatment for degenerative KOA. Of 80 patients who were initially enrolled, 72 patients completed the study. There was no significant difference between the primary and secondary outcomes of the two groups. We conducted an exploratory analysis of the subgroups based on a BMI of 25. Assessments of participants with a BMI of 25 or higher revealed no significant difference between the two groups. Assessments of participants with BMI lower than 25, after the 4^th^ week compared to that at baseline, showed progressive pain reduction and a decrease in rescue medicine consumption in the GCB group.

In terms of GCB safety, during the study, a total of 41 adverse events occurred: 24 cases in the GCB group and 17 cases in the placebo control group. There were two SAEs (1 hypertension and 1 back pain), all of which occurred in the placebo group and all recovered. GCB contains a cardiotoxic ingredient called “Aconiti Radix Processa.” Therefore, GCB should be prescribed by the TEAM physician with a regular laboratory test. [32]. However, a small amount of aconitine in GCB was clinically safe, and there were no adverse effects related to the toxicity of Aconiti Radix Processa during this study.

Although anti-inflammatory mechanism of GCB is not yet fully understood [13], it has been reported to inhibit the release of proinflammatory cytokines (IL-1*β*, IL-6, TNF-*α*, GM-CSF, and INF-*γ*), reduce the activity of inflammation-related mediators (iNOS, COX2, PGE2, and NO), and downregulate NF*κ*B and MAPK signaling. In addition, rhizomes of *Atractylodes lancea* (AL, Compositae, Chinese name: Cangzhu; Japanese name: Sou-ju-tsu) combined with GCB have been traditionally used in the treatment of digestive disorders, rheumatic diseases, and influenza in China, Korea, and Japan. The effect of AL has been attributed to anti-inflammatory properties resulting from downregulation of TNF-*α*, IL-8, IL-6, and PGE2 and gastric protective effects resulting from upregulation of EGF and TFF2 [33]. The anti-inflammatory effects of AL may alleviate knee pain and restore function by reducing systemic inflammatory conditions, including those of the gastrointestinal system, which is involved in the pathogenesis of KOA. Also, GCB contains a cardiotoxic ingredient called “Aconiti Radix Processa,” and it should be administered with caution. Aconiti Radix Processa contains aconitine, which has pericardial toxicity; however, a small amount of aconitine in GCB was clinically safe, and there were no adverse effects related to its toxicity during this study.

The clinical interest of this study was to explore the responsive subgroup to GCB in degenerative KOA patients. In subgroup analysis, GCB might be more effective in subjects with BMI below 25. However, as it is an exploratory analysis, we should be cautious while interpreting the data, considering multiple comparison bias. Pattern diagnosis, which not only considers symptoms of a patient but also the complexion and physique, is important when administering herbal medicine. In TEAM, various diagnostic methods have been used to administer herbal formulas, including a “cold-heat/deficiency-excess pattern” diagnosis [34–36]. GCB is a traditionally used herbal medicine for patients with pain and has been primarily prescribed for patients with poor nutrition and a skinny, cold intolerance tendency [37]. Individual drugs such as Cinnamomi Cortex, Atractylodes Lancea Rhizome, Zingiberis Rhizoma, and Aconiti Radix Processa have also been used to treat low vitality and cold intolerance pattern syndrome [37]. According to a previous study, the higher the BMI, the higher the tendency of the heat intolerance pattern, and the lower the BMI, the higher the cold intolerance pattern tendency [34]. According to a previous study, the risk of heat intolerance increased with BMI [34]. In addition, another study found that BMI is an important factor in determining the deficiency-excess pattern of the human body; the risk of the deficiency pattern also increased with lower BMI [35]. In a recent study using a prediction model, the BMI was an important item for predicting the “deficiency–excess” syndrome in TEAM practice [38]. We can anticipate that BMI will affect the therapeutic effect of herbal medicine. Based on these previous outcomes and the observations from our study, GCB is considered to be more effective in KOA patients with low BMI, cold intolerance, and deficiency patterns rather than those with high BMI and heat intolerance. Recent advances in molecular biology have also revealed that genes and metabolites are different than those resulting from the “cold and deficiency pattern” to the “heat and excess pattern” [39], that the prognosis of the disease is different from those identified by the “cold-heat/deficiency-excess pattern” diagnosis [40], and that the treatment targets are different by basics separated by the “cold-heat and deficiency-excess pattern” [41]. These studies demonstrated that appropriate treatment varies with patient characteristics or pattern diagnosis for the same diseases or symptoms. Subsequent studies should be conducted on the “cold pattern” or “deficient pattern” subjects through the pattern diagnosis on the “cold-heat and deficiency-excess pattern” to confirm the exploratory hypothesis of this study and the responses of the participants from the GCB group. Additionally, it is necessary to investigate the biomarkers that are predictive of clinical responses within the same diagnosis pattern group by applying the omics method.

We permitted participants to take painkillers (acetaminophen), as rescue medicine, only if they had intolerable discomfort due to knee pain. The comparison of analgesic doses in the GCB group and the control group showed that the doses were significantly lower in the GCB group than the control group. Current treatment guidelines recommend topical nonsteroidal anti-inflammatory drugs as an alternative and even first-line therapy for KOA management [42]. Since these drugs have side effects, including gastrointestinal bleeding or perforation, high blood pressure, and kidney failure, efforts have been ongoing for the discovery of safer alternatives [43, 44]. In previous studies, sole or combination therapy with herbal formulas reduced NSAID dosage and improved quality of life [45]. Similarly, GCB may be administered to patients who are taking medication for knee pain to reduce their doses and side effects and alleviate their symptoms more effectively. Furthermore, research on the effects of GCB when used as the sole therapy and an adjunct to conventional treatment is required. Consequently, this study may be considered as fundamental.

This study has several strengths. It is meaningful as the first clinical study on the use of GCB for treating degenerative KOA. This study minimized the nonspecific effects using controls placed on placebo. We continued with assessments after the course was completed, for long-term follow-up. In addition, we presented the possible responder of KOA patients to GCB. This study also had limitations. Since it is the first study on this topic, the sample size of 80 was small. In addition, the 4-week duration for taking GCB was insufficient for exploring the efficacy of long-term administration. The associated biomarkers were also not evaluated together, and pattern identification was not applied. We hope that future studies will explore mechanisms of GCB for degenerative KOA and its long-term effects. Furthermore, research is also required on the “cold/deficient pattern” syndrome differentiation, which was proposed in this study as a possible responder whose BMI is lower than 25. A research design using system biology biomarkers needs to be explored to determine if the “cold/deficiency pattern” group has a higher rate of treatment response than other groups and if there is a unique biomarker that is predictive of responses within the “cold/deficiency pattern” group.

## 5. Conclusion

The present study showed that four weeks of GCB intervention did not statistically significantly improve the knee pain, function, or quality of life of degenerative KOA patients compared to placebo. However, GCB reduced rescue medicine consumption. Also, it is estimated that a group of BMI 25 or less might be a responder group. They showed a significant reduction in pain intensity and rescue medicine consumption. Furthermore, research is needed to apply the system biology method with long-term follow-up.

## Figures and Tables

**Table 1:** Study design.

Assessment	Enrollment	Treatment phase			Follow-up phase
	Screening (−2 weeks∼day 0)	Visit 1 (0 weeks)	Visit 2 (2 weeks)	Visit 3 (4 weeks)	Visit 4 (8 weeks)

Informed consent	**○**				
Inclusion/exclusion criteria	**○**				
Vital signs and physical examination	**○**	**○**	**○**	**○**	**○**
Demographic characteristics	**○**				
Medical history	**○**				
Treatment expectancy questionnaire	**○**				
Blood test	**○**			**○**	
EKG	**○**			**○**	
Radiography of both knee	**○**				
Randomization		**○**			
VAS	**○**	**○**	**○**	**○**	**○**
K-WOMAC		**○**	**○**	**○**	**○**
EQ-5D		**○**	**○**	**○**	**○**
PGIC		**○**	**○**	**○**	**○**
Medication compliance			**○**	**○**	
Check rescue medicine and concomitant treatment			**○**	**○**	**○**
Safety assessment		**○**	**○**	**○**	**○**
Blinding test				**○**	
Medication administration		**○**	**○**		
Participants education	**○**	**○**	**○**	**○**	

VAS, visual analogue scale; K-WOMAC, Korean Western Ontario and Mcmaster Universities Osteoarthritis Index; EQ-5D, European Quality of life Five Dimensions questionnaire; PGIC, Patient Global Impression of Change. ^*∗*^Blood test: red blood cells (RBCs), white blood cell (WBCs), hemoglobin, hematocrit, platelets, erythrocyte sedimentation rate (ESR), C-reactive protein (CRP), aspartate aminotransferase (AST), alanine aminotransferase (ALT), gamma-glutamyl transferase (*γ*-GTP), total bilirubin, blood urea nitrogen (BUN), creatinine, and electrolytes (Na, K, and Cl).

**Table 2:** Composition and dose of GCB.

Name of herb	Dry weight (g)

Cinnamomi Cortex (桂皮)	4.0
Paeoniae Radix (芍藥)	4.0
Atractylodes Lancea Rhizome (蒼朮)	4.0
Zizyphi Fructus (大棗)	4.0
Glycyrrhizae Radix (甘草)	2.0
Zingiberis Rhizoma (生薑)	1.0
Aconiti Radix Processa (附子)	0.5
GCB, Gyejigachulbutang	7.5 g/day

**Table 3:** Demographics and baseline health characteristics (*N* = 80).

Characteristics		GCB group^†^	Placebo group^†^	*p* value^a^
Gender	Male	11 (27.50%)	9 (22.50%)	0.7968
	Female	29 (77.50%)	31 (77.50%)	
Age (year)		66.53 (63.95, 69.10)	65.38 (62.51, 68.24)	0.5472
Height (cm)		157.46 (155.15, 159.77)	158.00 (155.14, 160.87)	0.7652
Weight (kg)		62.33 (59.59, 65.07)	62.36 (59.37, 65.35)	0.9871
BMI (kg/m^2^)		25.11 (24.23, 25.99)	24.94 (24.00, 25.87)	0.7860
Job	Yes	33 (82.50%)	32 (80.00%)	0.9999
	No	7 (17.50%)	8 (20.00%)	
Exercise	Yes	28 (70.00%)	20 (50.00%)	0.1095
	No	12 (30.00%)	20 (50.00%)	
Exercise time (minute/week)		273.00 (213.50, 332.6)	207.50 (138.00, 277.00)	0.1464
X-ray				
K-L grade 2		31 (77.50%)	28 (70.00%)	0.5600
K-L grade 3		7 (17.50%)	11 (27.50%)	
K-L grade 4		2 (5.00%)	1 (2.50%)	
Morbidity period (month)		97.43 (74.19, 120.66)	91.35 (71.51, 111.19)	0.6887
Treatment expectancy		17.08 (16.12, 18.03)	17.00 (15.82, 18.18)	0.9207

^†^Data expressed either *n* (%) or 95% confidence interval. ^a^*p* value are from the independent two-sample *t*-test for continuous variables and from either the chi-squared test or Fisher's exact test for categorical variables. GCB, Gyejigachulbutang; BMI, body mass index; K-L grade, Kellgren–Lawrence grade.

**Table 4:** Treatment effect as measured at baseline, week 2, week 4, and week 8 (*N* = 80).

Characteristic	Baseline^†^	Week 2^†^	Mean difference^†^	*p* value^b^	Week 4^†^	Mean difference^†^	*p* value^b^	Week 8^†^	Mean difference^†^	*p* value^b^
VAS										
GCB	57.95 (52.16, 63.74)	52.55 (46.04, 59.06)	−5.40 (−7.92, −2.88)	**<0.0001** ^*∗*^	54.03 (47.33, 60.72)	−3.93 (−7.21, −0.64)	**0.0196** ^*∗*^	47.17 (40.00, 55.33)	−10.78 (−19.67, −1.90)	**0.0174** ^*∗*^
Placebo	60.30 (54.35, 66.25)	52.76 (45.74, 59.78)	−7.54 (−12.92, −2.17)	**0.0061** ^*∗*^	61.47 (53.79, 69.05)	1.12 (−6.15, 8.39)	0.7615	50.17 (43.91, 56.43)	−10.13 (−17.92, −2.33)	**0.0109** ^*∗*^
*p* value^a^	0.5688	0.7002			0.1881			0.6368		
K-WOMAC										
GCB	44.23 (37.89, 50.56)	41.30 (35.79, 46.81)	−2.93 (−5.02, −0.83)	**0.0064** ^*∗*^	41.40 (35.02, 47.78)	−2.83 (−4.80, 0.85)	**0.0054** ^*∗*^	40.61 (33.35, 47.87)	−3.61 (−8.86, 1.63)	0.1768
Placebo	46.73 (41.57, 51.88)	43.76 (38.71, 48.81)	−2.97 (−6.62, 0.69)	0.1114	43.76 (37.67, 49.82)	−2.96 (−8.46, 2.54)	0.2914	37.78 (31.46, 44.10)	−8.95 (−14.61, −3.29)	**0.0020** ^*∗*^
*p* value^a^	0.5375	0.7571			0.8599			0.2103		
EQ-5D										
GCB	0.73 (0.69, 0.77)	0.72 (0.68, 0.76)	0 (−0.02, 0.01)	0.5930	0.73 (0.68, 0.77)	0 (−0.02, 0.01)	0.7971	0.75 (0.72, 0.79)	0.03 (−0.01, 0.06)	0.0955
Placebo	0.71 (0.67, 0.75)	0.72 (0.69, 0.75)	0.02 (−0.02, 0.06)	0.3946	0.74 (0.71, 0.77)	0.04 (−0.01, 0.08)	0.1596	0.76 (0.73, 0.8)	0.06 (0, 0.11)	**0.0337** ^*∗*^
*p* value^a^	0.4594	0.6055			0.3395			0.5466		

^†^Data expressed as 95% confidence interval. ^a^*p* value was calculated from analysis of covariance with baseline score as a covariate. ^b^*p* value was calculated from the paired *t*-test. ^*∗*^Significant difference. GCB, Gyejigachulbutang; VAS, visual analogue scale; K-WOMAC, Korean Western Ontario and McMaster Universities Osteoarthritis Index; EQ-5D, European Quality of life Five Dimensions questionnaire.

**Table 5:** Analysis of participants with BMI 25 or more and BMI less than 25 (*N* = 80).

VAS	BMI 25 or more (*n* = 43)			BMI less than 25 (*n* = 37)		
	GCB group^†^	Placebo group^†^	*p* value^‡^	GCB group^†^	Placebo group^†^	*p* value^‡^

Baseline	56.52 (49.44, 63.60)	55.45 (47.08, 63.82)	0.8675	59.88 (49.29, 70.47)	65.15 (56.49, 73.81)	0.7951
Week 2	54.26 (45.55, 62.97)	52.57 (42.39, 62.75)		50.24 (39.40, 61.07)	52.95 (43.22, 62.67)	
Difference	−2.26 (−5.89, 1.06)	−2.88 (−6.13, 0.36)		−9.45 (−13.38, −5.92)	−12.20 (−15.03, 9.38)	
*p* value^a^	0.1808	0.0812		**<0.0001** ^*∗*^	**<0.0001** ^*∗*^	
Week 4	57.22 (48.35, 66.09)	56.40 (44.95, 67.86)	0.9434	49.71 (38.74, 60.67)	66.44 (57.07, 75.80)	**0.0239** ^*∗*^
Difference	0.70 (−3.61, 5.00)	0.95 (−4.24, 6.14)		−10.18 (−15.06, −5.30)	1.29 (−1.64, 4.21)	
*p* value^a^	0.7496	0.7166		**<0.0001** ^*∗*^	0.3857	
Week 8	48.33 (37.90, 58.77)	47.90 (38.04, 57.75)	0.9353	45.59 (32.46, 58.72)	52.45 (44.44, 60.46)	0.5772
Difference	−8.19 (−14.01, −2.37)	−7.55 (−13.20, −1.90)		−14.29 (−18.80, −9.79)	−12.70 (−16.73, −8.68)	
*p* value^a^	**0.0062** ^*∗*^	**0.0093** ^*∗*^		**<0.0001** ^*∗*^	**<0.0001** ^*∗*^	

^†^Data expressed as 95% confidence interval. ^‡^The mean difference was analyzed through analysis of covariance with the baseline score as covariant. ^a^*p* value was calculated from the paired *t*-test, ^*∗*^Significant difference. VAS, visual analogue scale. GCB, Gyejigachulbutang.

**Table 6:** Analysis of blind maintenance (*N* = 77).

Blind	GCB group	Placebo group	*p* value^a^

GCB group	15 (37.5%)	14 (37.8%)	0.0104^*∗*^
Placebo group	1 (2.5%)	9 (24.4%)	
Do not know	24 (60.0%)	14 (37.8%)	
New blind index^†^	0.35 (0.19, 0.51)	−0.14 (−0.39, 0.12)	

^†^Data expressed as 95% confidence interval. ^a^*p* value was calculated from the Fisher's exact test. ^*∗*^Significant difference. GCB, Gyejigachulbutang, rescue medicine consumption (post hoc analysis).

**Table 7:** Analysis of rescue medicine consumption.

VAS	GCB group^†^	Placebo group^†^	Mean difference^†^	*p* value^a^

Week 2	11.06 (total 188; *n* = 17)	18.00 (total 234; *n* = 13)	−6.94 (−15.83, 1.95)	0.1210
Week 4	5.41 (total 92; *n* = 17)	11.73 (total 176, *n* = 15)	−6.32 (−11.36, −1.25)	0.0162^*∗*^
Week 8	14.43 (total 202; *n* = 14)	17.00 (total 187, *n* = 11)	−2.57 (−15.04, 9.90)	0.6736

^†^Mean amount of rescue medicine consumption in patient who took rescue medicine (tablet); data expressed as 95% confidence interval. ^a^*p* value was calculated from the independent two-sample *t*-test. ^*∗*^Significant difference. GCB, Gyejigachulbutang.

## Data Availability

The data are available on request to the corresponding author..
